# Identification of patients with mood disorder following admission with hip fracture with a view to starting treatment and provide advice

**DOI:** 10.1192/bjo.2021.508

**Published:** 2021-06-18

**Authors:** Karla Louise Giles, Lisa Macpherson, Maria del Pilar Martin-Hernandez, Helen Wilson, Philip Hall, Keri Thompson, Sarah Bailey

**Affiliations:** 1 St Charles Hospital; 2 Royal Surrey County Hospital

## Abstract

**Aims:**

The aim of this quality improvement project is to improve identification and management of mood disorder in patients over 65 years admitted to Royal Surrey County Hospital (RSCH) with hip fractures by introducing a standardised assessment tool to guide appropriate interventions.

**Background:**

The signs of depression in the elderly can be subtle and often go unnoticed. The multidisciplinary team (MDT) at RSCH observed that low mood could negatively impact on a patient's recovery, affecting pain thresholds and leading to poor engagement with rehabilitation. Proactive identification and management of mood disorder is an important part of Comprehensive Geriatric Assessment but not routinely performed in patients with hip fracture admitted to RSCH.

**Method:**

Notes and discharge summaries of patients with hip fracture admitted over a four-month period were retrospectively reviewed to establish if patients were screened for low mood. A mood screening tool was chosen and implemented prospectively over a four-month period. Occupational therapists and junior doctors completed a Cornell Score for all patinets. Those identified with depression or probable depression were issued verbal advice, an information leaflet and follow-up arranged.

**Result:**

Ninety-eight patients were included in the retrospective cohort. No patients were formally identified as having depression or probable depression, and there was no indication that mood was considered or assessed at any point during admission. During the four-month prospective period, 90 patients were admitted to RSCH with hip fracture and 86 patients (96%) were screened for low mood. Four patients were excluded due to a terminal prognosis. Of the patients screened, 9% had major depression and 16% probable depression. Feedback from our occupational therapists and doctors was positive, with the tool being relatively easy to use in patients with or without cognitive impairment. Much of the assessment could be incorporated into their initial assessment or in gaining collateral history from next of kin. Anecdotally, considering patients psychological well-being had a positive impact on inpatient therapy sessions guided the MDT in supporting the patient appropriately.

**Conclusion:**

Implementation of a standardised and validated mood screening tool enabled us to identify that a quarter (25%) of the patients admitted following a hip fracture had, or probably had depression. This allowed us to intervene with simple measures such as verbal advice and an information leaflet and consider pharmacological intervention where appropriate.

